# Mortality Risk Factors for Pandemic Influenza on New Zealand Troop Ship, 1918

**DOI:** 10.3201/eid1612.100429

**Published:** 2010-12

**Authors:** Jennifer A. Summers, Nick Wilson, Michael G. Baker, G. Dennis Shanks

**Affiliations:** Author affiliations: University of Otago, Wellington, New Zealand (J.A. Summers, N. Wilson, M.G. Baker);; Australian Army Malaria Institute, Enoggera, Queensland, Australia (G.D. Shanks)

**Keywords:** Influenza, pandemic, viruses, New Zealand, infectious disease outbreak, troop ship, mortality, risk factors, military, historical review

## Abstract

We describe the epidemiology and risk factors for death in an outbreak of pandemic influenza on a troop ship. Mortality and descriptive data for military personnel on His Majesty’s New Zealand Transport troop ship Tahiti in July 1918 were analyzed, along with archival information. Mortality risk was increased among persons 25–34 years of age. Accommodations in cabins rather than sleeping in hammocks in other areas were also associated with increased mortality risk (rate ratio 4.28, 95% confidence interval 2.69–6.81). Assignment to a particular military unit, the field artillery (probably housed in cabins), also made a significant difference (adjusted odds ratio in logistic regression 3.04, 95% confidence interval 1.59–5.82). There were no significant differences by assigned rurality (rural residence) or socioeconomic status. Results suggest that the virulent nature of the 1918 influenza strain, a crowded environment, and inadequate isolation measures contributed to the high influenza mortality rate onboard this ship.

## Abstract 2

TOC summary: Crowding and ventilation problems contributed to an increased risk of death.

To plan and prepare appropriately for future influenza pandemics, public health authorities need to better understand the epidemiology of previous pandemics. Much remains obscure about the epidemiology of the influenza pandemic of 1918–19, the spread of which depended on the transportation of large numbers of troops during World War I.

Pandemic influenza outbreaks among closed military populations are problematic and sometimes show high mortality rates. Reports on this topic have been published. These include descriptions of 1918 pandemic outbreaks in U.S. and Australian troop and civilian ships in 1918–19 (1–5), descriptions of 1918 pandemic outbreaks in military camps in the United States, the United Kingdom, and New Zealand (2,3,6–8), and more recent influenza outbreaks onboard naval and civilian ships (9–12).

Some studies have investigated specific risk factors for death from the 1918 pandemic. Evidence has shown that lower socioeconomic status increased mortality risk (13,14) and that young adults, for as-yet-unexplained reasons, had disproportionately higher mortality rates (13–16). Rural living versus urban living is another risk factor that has been investigated and has showed conflicting results (17–20). Lower mortality rates were observed among seasoned troops (>6 months experience) compared with newly recruited troops, possibly because of previous exposure to respiratory pathogens in seasoned troops (8,21,22).

The purpose of this study was to examine the 1918 outbreak on His Majesty’s New Zealand Transport (HMNZT) Tahiti (Figure 1) and to identify mortality risk factors among persons onboard. During and after World War I, HMNZT Tahiti made numerous trips, transporting reinforcements and supplies from New Zealand to Europe, and bringing home New Zealand troops (Figure 2). On July 10, 1918, HMNZT Tahiti departed New Zealand with the 40th Reinforcements, a unit that consisted largely of infantry replacements. The voyage across the Indian Ocean and around the Cape of Good Hope was uneventful. HMNZT Tahiti was to join a convoy in Freetown, Sierra Leone, before heading to England. Upon reaching Freetown, reports of disease ashore resulted in all ships in the convoy being quarantined at port (7,25). However, a conference was attended by captains and wireless operators from every ship in the convoy onboard the His Majesty’s Ship Mantua. The Mantua had experienced an influenza outbreak onboard 2 days after leaving the United Kingdom on August 1, 1918, and is thought to have been responsible for bringing the second wave of the 1918 pandemic to western Africa from England (5,26).

**Figure 1:**
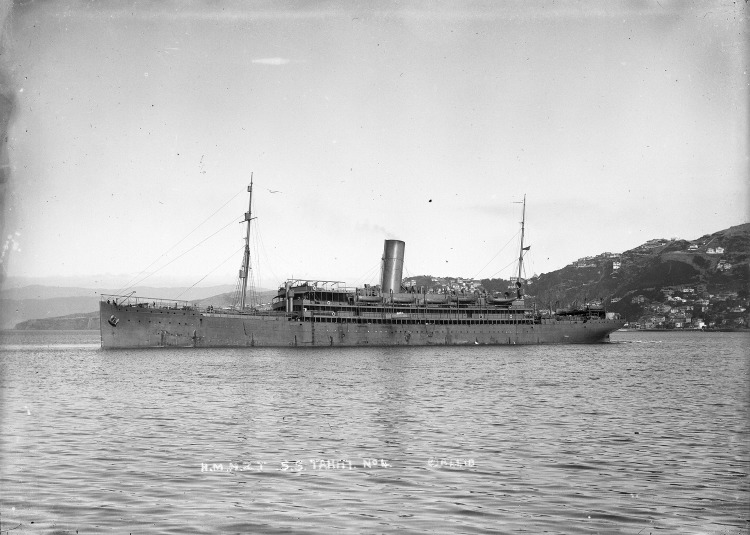
His Majesty’s New Zealand Transport Tahiti in Wellington Harbor (c. 1914–1919). Photograph was taken by an unidentified photographer (23).

**Figure 2:**
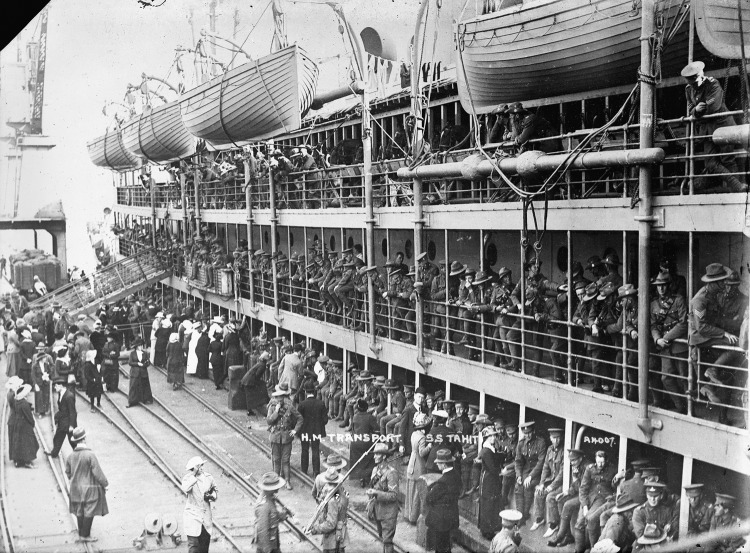
His Majesty’s New Zealand Transport Tahiti with World War I troops alongside a wharf (c. 1915). This photograph was presumably taken in a Wellington, New Zealand, wharf, given the gauge of the railway tracks and the crane type. Photograph was taken by David J. Aldersley (24).

HMNZT Tahiti left Freetown on August 26, 1918, as part of the convoy after being resupplied by local workers (who were another possible source of infection with the new pandemic influenza strain). On the day of sailing, influenza case-patients began to be admitted to the onboard hospital. Over the next few weeks of the voyage, influenza developed in >1,000 of the 1,217 persons onboard (25). By the time HMNZT Tahiti reached Plymouth, England, on September 10, 1918, a total of 68 men had died onboard the ship (23,27). Eight other men and 1 nurse who had been on the ship died of influenza in England. HMNZT Tahiti, the worst affected ship in the convoy, was referred to as the death ship, and a Court of Inquiry was held to investigate this outbreak.

## Historical Context and Mortality Data

Historical information was obtained from the official report of the outbreak held in Wellington from Archives New Zealand (27), the Inquiry Report from the Transport Epidemic Committee to the House of Representatives of New Zealand, dated December 9, 1918, and the written account of Colonel E.J. O’Neill as officer commanding the 40th Reinforcements (25). Individualized data on all military personnel on the July 1918 sailing of HMNZT Tahiti recorded in the Cenotaph database were obtained from the Auckland War Memorial Museum (28). An electronic dataset (Roll-of-Honor) covering all deaths among New Zealand military personnel during World War I was obtained from Peter Dennis (Australian Defence Force Academy, University of New South Wales, Canberra, Australian Capital Territory). The Roll-of-Honor and Cenotaph databases were matched to identify persons onboard HMNZT Tahiti whose death from the disease had been listed. The precise cause of death was only reported in the Cenotaph database for 3 of 77 case-patients and was recorded as influenza or pneumonia. One death recorded as a drowning was included because a recently published study showed that the drowning occurred when a febrile soldier aboard HMNZT Tahiti threw himself into the sea (29).

## Demographic Data

Few records in the Cenotaph database included age data (n = 16). Therefore, the age of those persons aboard HMNZT Tahiti during the voyage was determined for 864 persons (77.4%) on the basis of the soldier’s date of birth from the Roll of Casualties held at Archives New Zealand (30) and an online database for births, deaths, and marriages in New Zealand (31).

Preenlistment occupations were coded for occupational class as per a New Zealand–specific system for historical classification of occupational class (32) by using 1919 codes and a website (http://caversham.otago.ac.nz/electors/erform.php). This classification provided results such as laborer (code 9) and company manager (code 1). If an occupation was not listed, the classification for a different census year (e.g., 1924) or the closest match (e.g., orchardist to gardener) was used. Only 13 (1.16%) records had no occupation or could not be coded.

All records with an enlistment address (n = 15) or next-of-kin address (n = 1,088) were given a rurality score on the basis of the rural/urban classification in a previous study (17). Because some (n = 167) of these addresses could not be readily classified, further work to assign a rurality score was conducted by using an estimate of likely population levels in 1918 and Google Maps (33). A scoring system for grading rurality was developed on the basis of occupation and address. All occupations were ranked for likelihood of being a rural-based job: definitely rural = 4 (e.g., farmer); probably rural = 2 (e.g., a fence builder or other occupations); and 0 (e.g., accountant). The final rurality index ranged from 0 (urban) to 8 (rural), which is the combined score of the address rurality score and the occupation rurality score.

## Military Data

Military rank was divided into categories on the basis of a key military text (34) and other available information regarding the New Zealand Expeditionary Force. These categories were officers, noncommissioned officers, healthcare workers, and others. The Cenotaph database information was used to classify persons by their military units. Most persons onboard HMNZT Tahiti belonged to specific companies within the 40th Reinforcements. All military personnel (n = 30) with embarkation dates before HMNZT Tahiti sailed on July 10, 1918, were identified as persons with previous military experience >1 month of service. The first embarkation date was used to estimate months in military service.

## Statistical Analyses

The association of demographic, socioeconomic, and other variables with mortality risk was analyzed by using univariate and multivariate analyses. In multivariate logistic regression analyses, 1 model considered the demographic and sociodemographic factors, and the more fully adjusted model also included military unit. All analyses used Stata version 10 (StataCorp LP, College Station, TX, USA).

## Total Number of Cases

**Figure 3:**
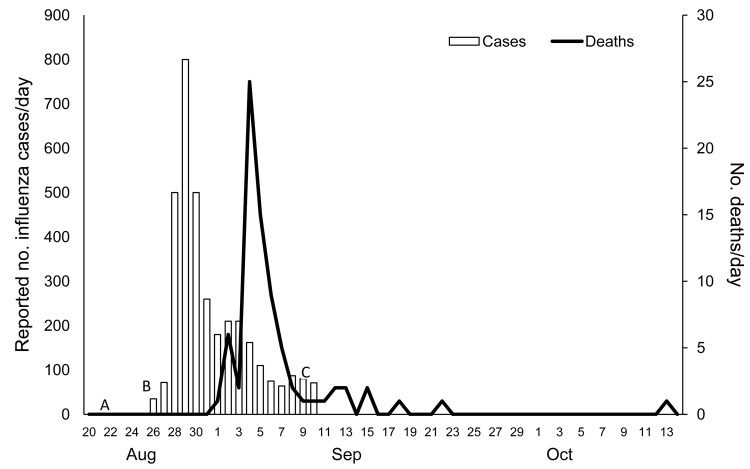
Cases of influenza and mortality rates for persons aboard His Majesty’s New Zealand Transport (HNZMT) Tahiti during an outbreak of pandemic influenza, 1918. Reported cases of influenza are approximate and the definition of a case was not precisely described. A, August 22, 1918, HMNZT Tahiti arrives in Sierra Leone; B, August 26, 1918, HMNZT Tahiti leaves Sierra Leone; C, September 10, 1918, HMNZT Tahiti arrives in England (subsequent deaths occurred in hospitals in England).

The total number of persons onboard HMNZT Tahiti at the time of the outbreak was 1,117 military personnel plus 100 crew (total 1,217 persons). This total included 6 deserters (who embarked in New Zealand but left the ship before the outbreak) but it was not possible to identify these persons and remove them from the dataset. The outbreak onboard reached its illness peak on August 29, 1918, and the peak number of deaths (20) occurred on September 4 (Figure 3). The Inquiry Report showed that the military commander estimated that 800 were sick on the peak day (on the basis of those who did not have breakfast and those who had duties caring for the sick) of the outbreak, and the overall mortality rate was 68.9 persons/1,000 population (25).

## Age Patterns and Mortality Rates

**Figure 4:**
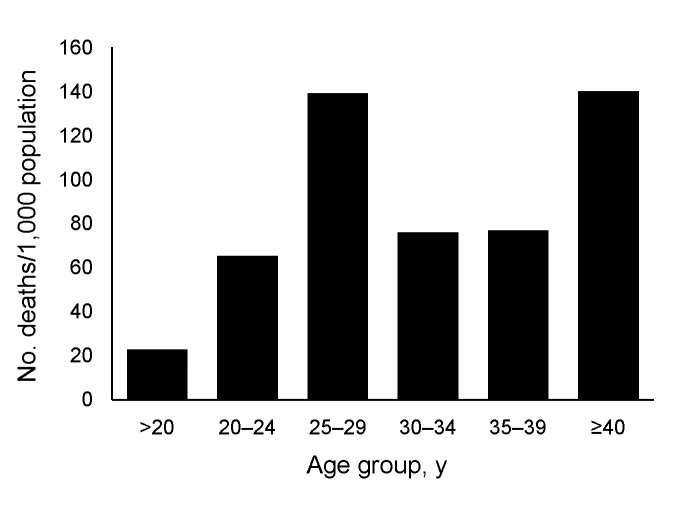
Mortality rates for persons aboard His Majesty’s New Zealand Transport Tahiti, by age group, during an outbreak of pandemic influenza, 1918.

The average age of those onboard HMNZT Tahiti was 26.7 years. Those >40 years of age (the smallest age group) had the highest mortality rate (140 persons/1,000 population (Figure 4). Ages were grouped into larger groups than shown in Figure 4 for further analysis. The mortality rate for persons 25–34 years of age was 108.1 persons/1,000 population, which was higher than that for persons 20–24 years of age (70 persons/1,000 population) and was higher than that for all other age groups combined (crude rate ratio [RR] 1.80, 95% confidence interval [CI] 1.08–2.92).

## Military Rank

Officers had the highest mortality rate among military personnel (83.3 persons/1,000 population). However, because only 1 officer died, this result was not significant when compared with the rates for noncomissioned officers and also the rate for all other ranks combined.

## Occupation and Rurality

No variations in the mortality rates were found for different occupational classes, rural occupations, and rurality of address. Additionally, no differences in mortality rates could be attributed to rurality scores (Table 1).

**Table 1:** Mortality rates during pandemic influenza outbreak, by rurality score, aboard His Majesty’s New Zealand Transport Tahiti, 1918*

Rurality score†	No. deaths	Mortality rate/ 1,000 persons	Crude mortality rate ratio (95% CI)
0 (urban)	32	81.0	1.0 (reference)
1–2	18	60.6	0.75 (0.43–1.31)
3–4	12	60.3	0.74 (0.39–1.41)
5–6	7	59.8	0.73 (0.33–1.63)
7–8	8	88.9	1.10 (0.52–2.30)

*CI, confidence interval. †Calculated by using occupation and address. Highest score was rural occupation plus rural address.

## Crowding and Military Unit

On the basis of postoutbreak data in archival sources (25,27), the mortality rate by types of accommodation could be analyzed. This comparison showed a higher mortality rate for persons in cabins with bunks (39/267, 146.1 persons/1,000 population) than for persons in other areas in which hammocks were used (28/820, 34.1 persons/1,000 population) (25). This difference was significant (crude RR 4.28, 95% CI 2.69–6.81).

The 8 military units onboard HMNZT Tahiti (40th A, B, C, and E companies, 40th Field Artillery, 40th all groups, Medical Corps and Nursing, and all other groups) were housed separately. Only the 40th Field Artillery, which had a mortality rate of 152.4 persons/1,000 population, had a significantly increased mortality rate (crude RR 2.72, 95% CI 1.16–6.36). Anecdotal evidence in the Inquiry Report suggests that this unit was housed in cabins.

## Military Experience

No significant difference in mortality rates was found between persons with military experience and those without experience. Numbers were too small to assess whether the number of months in military service was associated with mortality risk in this outbreak.

## Multivariate Analyses

Two logistic regression models were used to analyze risk for death among those onboard HMNZT Tahiti (Table 2). In the more fully adjusted model (model 2), age was independently associated with increased mortality risk. Being in the Field Artillery (versus all other military units) was also independently associated with increased mortality risk (adjusted odds ratio 3.04, 95% CI 1.59–5.82). Military rank, occupational class, and rurality were not associated with mortality risk in either model.

**Table 2:** Multivariate analyses of risk for death during pandemic influenza outbreak onboard His Majesty’s New Zealand Transport Tahiti, 1918*

Variable	Model 1: demographics and sociodemographics†		Model 2: model 1 plus military unit‡

aOR (95% CI)	p value	aOR (95% CI)	p value
Demographic and sociodemographic					
Age, continuous	1.03 (1.00–1.06)	0.071		1.04 (1.00–1.07)	0.025
Military rank, officer plus NCO vs. all other ranks§	0.51 (0.24–1.12)	0.093		0.49 (0.23–1.08)	0.077
Occupational class based on prewar occupation, groups 7–9 vs. groups 1–6¶	0.72 (0.44–1.18)	0.196		0.83 (0.50–1.38)	0.468
Rurality score, continuous	0.97 (0.87–1.08)	0.558		1.00 (0.90–1.12)	0.943
Other					
Military unit, 40th Reinforcements New Zealand Field Artillery vs all other units combined				3.04 (1.59–5.82)	0.001

*CI, confidence interval; aOR, adjusted odds ratio for death during the outbreak; NCO, noncommissioned officer. †Hosmer-Lemeshow χ^2^ 8.47, degrees of freedom 8, p = 0.389. ‡Hosmer-Lemeshow χ^2^ 13.06, degrees of freedom 8, p = 0.110. §Healthcare workers were included in other ranks. ¶Groups 1–6 indicate higher status occupations.

## Conclusions

A shipboard epidemic of influenza resulted when persons onboard HMNZT Tahiti were infected in Sierra Leone. The Inquiry Report states that “The disease appeared in severe and epidemic form on August 26.” (25). The date coincides with the outbreak of the more severe second wave of the pandemic in western Africa (26). During the earlier stages of the voyage, the report states that “the number of sick has been remarkably low” (25).

The estimated cumulative incidence of pandemic influenza (90%) on HMNZT Tahiti was similar to the highest levels on other ships from Australia, such as the Ooma (88%) (1,4), and much higher than the estimated cumulative incidence of one of the worst affected US troop ships, USS Leviathan, which had a cumulative incidence of 20% (2,3). One of the highest reported mortality rates on any ship during the pandemic was that of the Atua, which sailed November 2, 1918 (98.2 persons/1,000 population) (1), which was similar to that observed for HMNZT Tahiti, although the Atua was a much smaller ship that was carrying 163 persons.

The nature of the sleeping area (cabins with bunks rather than hammocks) was associated with increased mortality risk in this outbreak. The Court of Inquiry stated that one of the main reasons for the high mortality rate in this outbreak was poor ventilation systems onboard HMNZT Tahiti (25). The system of closing port holes at night and during danger periods (bad weather and U-boats in the water) and ineffective wind sails resulted in insufficient ventilation to sleeping areas. It was recommended that some form of artificial ventilation be introduced in the future. Anecdotal evidence from troops interviewed after the outbreak reported that the cabins had poorer ventilation than other accommodations (25). This situation may have been caused by makeshift conversions of cabins on HMNZT Tahiti, which potentially blocked ventilation, even though the space allotted to each person was approximately equivalent in both types of accommodation (≈110 ft^3^ of airspace/person). Good ventilation may play a role in preventing or limiting spread of viral influenza by airborne transmission. One study of an isolated influenza outbreak onboard a commercial airliner suggested that an inoperative ventilation system was the cause of the high attack rate (35). Additionally, 1 study reported that open-air treatment was associated with reduced morality rates during the 1918 pandemic (36). However, more recent analysis of viral influenza transmission suggests that the infection is transmitted primarily by contact, followed by droplets, and to a lesser extent by airborne transmission (37,38).

Military personnel assigned to the 40th Reinforcements Field Artillery had a higher risk of dying from pandemic influenza than any other military unit on HMNZT Tahiti. Evidence from the inquiry suggests that all Field Artillery personnel were lodged in cabins (25). However, the Field Artillery personnel were unlikely to be the only unit placed in the cabins, given the numbers in the inquiry.

Although the inquiry found that HMNZT Tahiti was no more crowded than other similar troop ships, it was originally fitted for ≈650 passengers and crew (39), noticeably fewer than the 1,217 persons onboard during the July 1918 sailing. This crowding was caused by shipping shortages during World War I, which led to placing as many troops onboard a ship as possible. Isolation measures onboard HMNZT Tahiti, such as clearing deck space for temporary hospitals, were insufficient because the number of patients exceeded the capacity of the onboard hospital (25). A crowded environment and inadequate isolation appear to have exacerbated the influenza outbreak, enabling transmission of influenza virus through contact and droplets. These findings serve as a reminder to healthcare planners that the effects of an influenza outbreak within an institutionalized population, such as in hospitals, prisons, and ships, can be devastating without proper preparation beforehand to deal with the variety of potential transmission routes.

Older age was independently associated with increased mortality risk (by logistic regression). This finding was largely reflected in increased risk among persons 25–34 years of age than in persons <25 years of age. This finding is consistent with those of previous research (13–15) and with the total New Zealand population, in which the worst affected group was 30–34 years of age, which had a mortality rate of 15.5 persons/1,000 population (7). This rate is less than one fourth of the rate on HMNZT Tahiti. This difference may have been caused by crowding, with those onboard HMNZT Tahiti being exposed to higher infective doses of influenza virus or bacterial infections (e.g., *Streptococcus pneumoniae*). Persons onboard HMNZT Tahiti may also have not been exposed to the first wave of the pandemic, and therefore had no immunity to the new pandemic strain (8) because there is no evidence of a first pandemic wave in New Zealand before the July 1918 sailing of HMNZT Tahiti (7).

The medical and nursing personnel were overwhelmed by the mass casualty event caused by the influenza outbreak; many of them were incapacitated by illness when they were most needed. The use of strychnine, digitalis, and alcohol as stimulants for treating sick personnel onboard may have adversely affected mortality rates, but it is unlikely that any of the medications available in 1918 would have changed the outcome for most soldiers. Injections of an unspecific mixed catarrhal vaccine were given in the weeks before the outbreak (27), but what affect, if any, this vaccine may have had is unknown. Nevertheless, another study during this period found that a possibly similar vaccine, also described as a mixed catarrhal vaccine, could have had a favorable affect on influenza-related mortality rates (40).

Socioeconomic status and military rank did not appear to effect mortality rates. Additionally, lower occupational status was not related to higher mortality rates, which suggested that any potential differences in nutritional or health status before embarkation or during the voyage did not play any major role in mortality risk. Classifications of rurality by using preenlistment occupation, address, and rurality score did not show any differences in mortality rates. New recruits (first embarkation) were just as likely to die during the outbreak as seasoned troops, which is not consistent with results of previous research (8,21,22). However, the numbers of experienced soldiers were small in this particular outbreak.

There are many limitations in studying past events because of transcription and other recording errors. Military data were, understandably, never designed to capture detailed epidemiologic information. The lack of a proper case definition and only approximate estimates of case numbers in this outbreak limit their value for estimating epidemiologic parameters such as reproduction number. Use of preenlistment address and next-of-kin address as proxies for rurality may not give an accurate estimate of the geographic exposure of a person. The use of preenlistment occupation as a measure of socioeconomic status is also problematic because many persons may have been assigned to particular reserved occupations for the war effort, which did not reflect their prewar occupation. Conscripted men aboard HMNZT Tahiti may not have been representative of those remaining in New Zealand but they would have had to pass minimum medical standards to be in the military.

The outbreak on HMNZT Tahiti likely represents a worst-case scenario in which nonimmune soldiers were intensively exposed to a highly pathogenic virus while experiencing crowding and ineffective isolation measures. Perhaps the best use of the tragic story of HMNZT Tahiti is as a reminder that although the influenza pandemic that began in 2009 was relatively mild, influenza is capable of causing devastating mass casualties, especially in closed and crowded populations.
